# Generation of Quality Hit Matter for Successful Drug Discovery Projects

**DOI:** 10.3390/molecules24030381

**Published:** 2019-01-22

**Authors:** Jóhannes Reynisson

**Affiliations:** School of Chemical Sciences, University of Auckland, Private Bag 92019, Auckland 1142, New Zealand; j.reynisson@auckland.ac.nz

A drug discovery project needs a number of components for its success. From the point of view of many medicinal chemists, tractable hit matter is one of the most important parts in such a project. Without quality hits the projects grind to a halt and no further progress is possible, no matter how promising the modulation of the said biological target is for a novel therapeutic approach. This is obviously a very frustrating experience for all of the scientists involved and often the projects are simply discontinued. Perhaps borne out of this frustration an array of different screening approaches have been developed to mitigate the problem of no or poor quality hit matter, i.e., the more techniques available for hit generation the better since each approach has its strengths and weaknesses. In this special issue “Hit Generation and Verification for Novel Lead Compounds” (https://www.mdpi.com/journal/molecules/special_issues/novel_lead_compounds), the focus was on this crucial part of the drug discovery pathway and it is pertinent to keep in mind that the chain is as strong as its weakest link.

In the special issue a range of different hit generation methods were reported such as the marriage of the classical methods of natural products with structure based virtual screening, which were successfully reported to identify cannabinoid receptor 1 inverse agonists for treating obesity [1] and the generation of potent inhibitors of tyrosyl-DNA phosphodiesterase 1, a promising anticancer target [2]. A fragment based screen was reported against the FIXa target for blood anticoagulation [3], high content screen using zebrafish for cardiovascular issues focusing on the Fgf/Ras/Mapk activity was reported [4] and finally an in vitro/in silico method was introduced for the modulation of the Partial PPARγ receptor for the treatment of diabetes [5]. Additionally, more classical structural activity relationships studies were reported such as cationic non-peptic small molecules as membrane disruptors as antimicrobial agents [6] and the synthesis of proanthocyanidin derivatives as generally interesting bioactive compounds [7]. Lastly, two reports on the anticancer compound class thieno [2,3-*b*] pyridines were introduced where two major issues of small molecular drug discovery were addressed, the lack of aqueous solubility [8] and the identification of the molecular targets modulated by the molecules [9]. In the reported projects either natural products, and their derivatives, or synthetic small molecules are used as screening collections, i.e., very different regions of chemical space are explored. In general, the concept of chemical space is now well established and many researchers involved in drug discovery projects apply these ideas [10,11]. Traditionally, the volume of chemical space is reduced by molecular descriptors, such as molecular weight and lipophilicity (log P), and undesirable chemical moieties where molecules are excluded from further consideration, shown graphically in Figure 1 [12,13,14,15,16].

Known Drug Space (KDS) is a concept used to navigate chemical space to identify biologically benign volumes of small molecules as potential drug candidates [18,19,20]. E.g., frequency of atoms types and different substitution patterns can be derived and used as designing concepts [21,22,23,24]. KDS is defined as all small molecules in clinical use [25]. It has been shown that KDS has wider parameters in terms of both molecular descriptors and unwanted molecular moieties, resulting in a larger volume in chemical space compared to drug-like compounds, as shown in Figure 1 [26,27,28]. It is therefore clear that a more focused definition of biologically active compounds based on their physicochemical parameters would benefit the identification of quality hit matter enormously. This would have a beneficial knock-on effect on the drug discovery pathway where many of the problems of bio-incompatibility will simply not be encountered. Currently, novel molecular descriptors derived from the density functional theory are being developed as well as an index, Known Drug Index, to improve our navigational skills in chemical space [17,29,30]. 

## Figures and Tables

**Figure 1:**
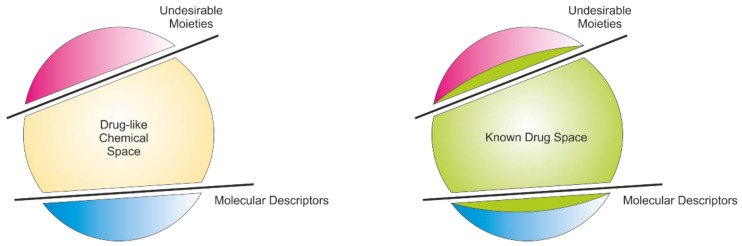
A graphical representation of chemical space reduced with undesirable moieties and molecular descriptors in defining drug-likeness. Known drug space occupies larger volume in chemical space [17].
